# A Novel IGBT with SIPOS Pillars Achieving Ultralow Power Loss in TCAD Simulation Study

**DOI:** 10.3390/mi15060759

**Published:** 2024-06-05

**Authors:** Song Yuan, Zhaoheng Yan, Yanzuo Li, Ying Wang, Qifan Liu, Xinbin Zhan, Xi Jiang, Yanjing He, Xiaowu Gong

**Affiliations:** 1 The Key Laboratory of Ministry of Education for Wide Bandgap Semiconductor Materials and Devices, School of Microelectronics, Xidian University, Xi’an 710071, China; syuan@xidian.edu.cn (S.Y.); zhyan_2@stu.xidian.edu.cn (Z.Y.); liyanzuo@stu.xidian.edu.cn (Y.L.); ywang1@stu.xidian.edu.cn (Y.W.); qifanliu@stu.xidian.edu.cn (Q.L.); xbzhan@stu.xidian.edu.cn (X.Z.); hyj@xidian.edu.cn (Y.H.); xwgong@xidian.edu.cn (X.G.); 2 The Guangzhou Institute of Technology, Xidian University, Guangzhou 510555, China

**Keywords:** Insulated Gate Bipolar Transistor (IGBT), Semi-Insulating POly-crystalline Silicon (SIPOS), Switching Loss

## Abstract

A novel insulated gate bipolar transistor with Semi-Insulated POly Silicon (SIPOS) is presented in this paper and analyzed through TCAD simulation. In the off state, the SIPOS-IGBT can obtain a uniform electric field distribution, which enables a thinner drift region under the same breakdown voltage. In the on state, an electron accumulation layer is formed along the SIPOS, which can increase the injection level of the “PiN region” in the device, and the carrier concentration in the drift region is also increased due to the charge balance effect. Moreover, the SIPOS-IGBT can achieve a quick and thorough depletion in the drift region during the turn-off transient, which can greatly reduce the turn-off loss of the SIPOS-IGBT. These advantages improve the tradeoff between the conduction and switching losses. According to the simulation results, the SIPOS-IGBT obtained a 58% lower turn loss than that of a field-stop (FS) IGBT and 30% lower than an HK-IGBT with the same on-state voltage.

## 1. Introduction

Insulated Gate Bipolar Transistors (IGBTs) are widely used in various power-switching applications for combining the best features of the BJT and MOSFET structures. However, as the key characteristics, the power losses of power devices are usually discussed, and IGBTs also face a crucial tradeoff between the on-state voltage (V_on_) and turn-off loss (E_off_) [1].

Field-stop (FS) IGBTs are widely used structures in current commercial products that can achieve low Von and low Eoff values by using a thinner drift region and specially designed trench gates [2]. Later, IEGT and CSTBT were proposed and applied in FS-IGBTs to improve the performance of the devices [3,4,5]. To further improve the tradeoff between the conduction loss and the switching loss, a superjunction structure was also applied in an IGBT, which could offer an ultralow turn-off loss during switching [6,7,8,9,10,11,12,13,14]. Even though fabrication processes such as deep trench refilling and multi-epi have been fully developed, the problem of charge imbalances due to the process deviation is still severe [15,16,17]. Another way to improve IGBT performance is by introducing a high-relative-permittivity (high-k or HK) dielectric into the drift region; HK-IGBTs can obtain a similar performance to SJ-IGBTs without the charge imbalance problem [18,19,20,21]. However, most HK materials are incompatible with the Si IGBT fabrication process, and the electric field peak at the bottom of HK-IGBTs may lead to reliability problems during the switching process [22,23]. 

In this article, a novel IGBT with semi-insulated poly silicon (SIPOS) introduced in the drift region is proposed and analyzed by simulation. With its uniform electric field distribution and electron accumulation layer, the SIPOS-IGBT can provide a better tradeoff between Von and E_off_ compared with a trench FS-IGBT and HK-IGBT; in addition, no charge imbalance or process compatibility problems exist with this device [24,25,26].

## 2. Device Structure and Mechanism

**Figure 1:**
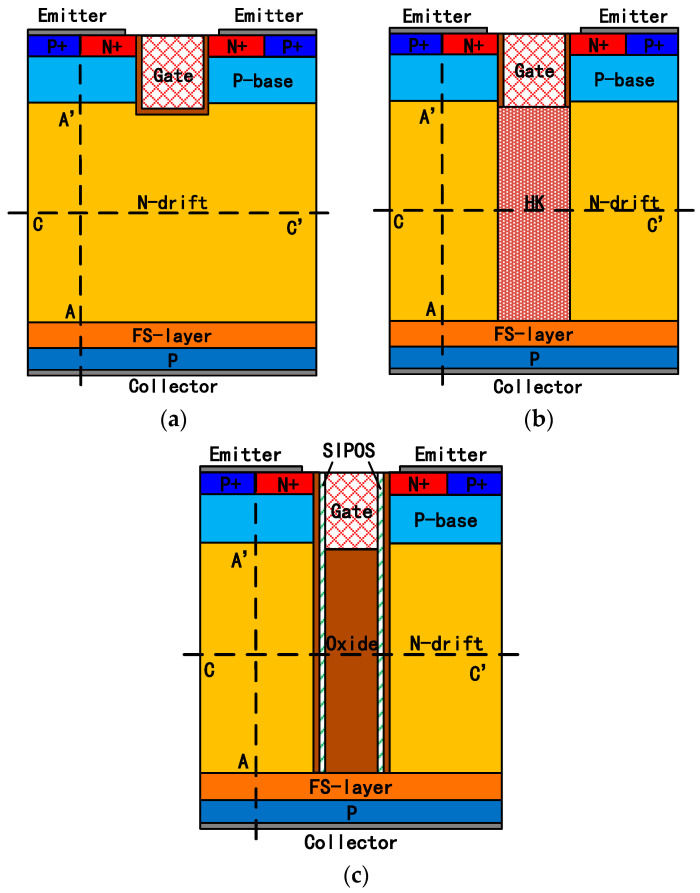
Schematic cross-sections of the devices. (**a**) FS-IGBT, (**b**) HK-IGBT, and (**c**) proposed SIPOS-IGBT.

Figure 1 illustrates the structures of an FS-IGBT, HK-IGBT, and the proposed SIPOS-IGBT. In Figure 1b, the HK pillar is alternated with the N-drift region. In Figure 1c, in the SIPOS-IGBT, two thin SIPOS pillars are located under the trench gate, and between the two SIPOS pillars is the deposed dielectric material (in this paper, it is oxide). Both the HK layer and SIPOS layer are in contact with the gate electrode and FS layers.

SIPOS is usually used as a resistive field plate material for its high resistivity, which leads to uniform electric field distribution along the SIPOS layer. So, the electric field distribution in the drift region of the SIPOS-IGBT is also modulated to be uniform, which enables a thinner drift region like a superjunction structure or HK structure. Moreover, an electron accumulation layer can be formed along the SIPOS pillar during the on state [27,28,29], and in the SIPOS-IGBT, the accumulation layer increases the injection efficiency of the anode of the “PiN region” in the device, so the total carrier concentration in the drift region is increased. With the effects mentioned above, the Von value is significantly reduced. In the off state, the SIPOS layer facilitates the depletion of the drift region like a superjunction structure or HK structure, and the turn-off time is accelerated, leading to ultralow turn-off loss.

## 3. Results and Discussion

In this section, the proposed SIPOS-IGBT is investigated and compared with an FS-IGBT and HK-IGBT by using a TCAD simulation. The SIPOS was defined as a semiconductor material, and the main physics models used in the simulations were Mobility (DopingDep HighFieldSat Enormal), EffectiveIntrinsicDensity (OldSlotboom), Recombination (SRH (DopingDep), and Auger Avalanche (Eparal)) [30]. The device parameters used in the simulation are listed in Table 1.

### 3.1. Breakdown Characteristics

**Figure 2:**
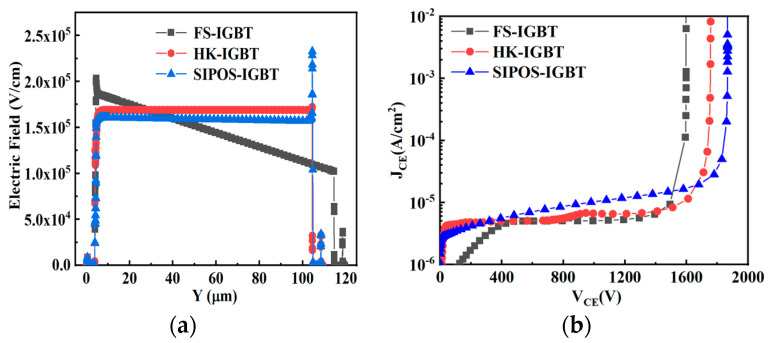
(**a**) Electric field distribution along A-A’; (**b**) breakdown characteristics of FS-IGBT, HK-IGBT, and SIPOS-IGBT.

Figure 2a shows the electric field distributions for the FS-IGBT, HK-IGBT, and SIPOS-IGBT. The electric field distribution in the HK-IGBT and SIPOS-IGBT was more uniform than in the FS-IGBT. This means that the FS-IGBT needs a thicker drift region to achieve the same breakdown voltage compared with the HK-IGBT and SIPOS-IGBT. Furthermore, a high electric field peak appeared at the bottom corner of the HK pillar in the HK-IGBT due to the electric flux crowding effect in the HK material. This electric field peak may lead to premature breakdown under high reverse-biased conditions or dynamic avalanches during the switching progress [22,23]. So, with its flat electric field distribution, the SIPOS-IGBT always achieved the highest breakdown voltage compared with the FS-IGBT and HK-IGBT, as shown in Figure 2b.

**Figure 3:**
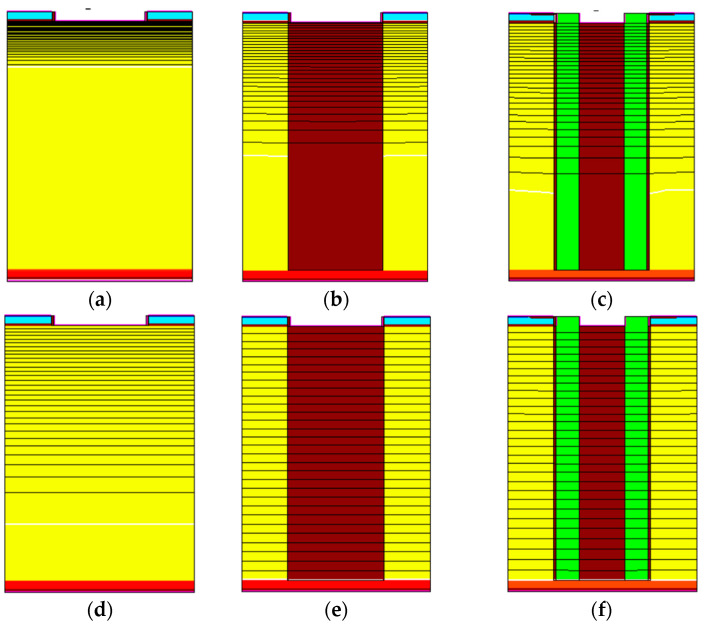
Electric potential distribution of (**a**,**d**) FS-IGBT, (**b**,**e**) HK-IGBT (brown pillar in the middle is HK material), and (**c**,**f**) SIPOS-IGBT (brown pillar is oxide, green pillars are SIPOS) under a reverse voltage of 20 V (**a**–**c**) and 300 V(**d**–**f**).

Figure 3 shows the potential distribution under the same reverse voltage; with a low reverse voltage (20 V), the SIPOS-IGBT and HK-IGBT offered a wider depletion region than the FS-IGBT. When the reverse voltage was higher than 300 V, the HK-IGBT and SIPOS-IGBT were fully depleted. This phenomenon will enhance the depletion of the drift region during the turn-off transient [20]. In contrast, there was still a neutral region existing in the FS-IGBT.

### 3.2. On-State Characteristics

**Figure 4:**
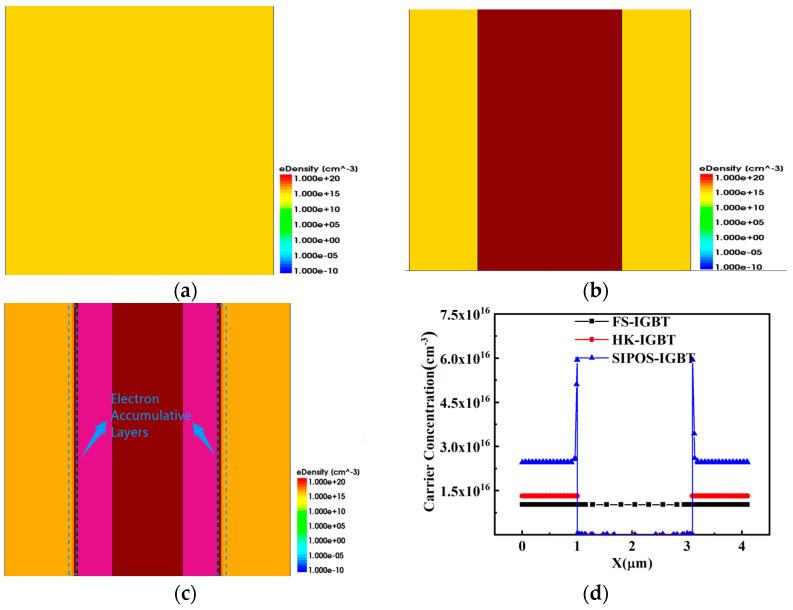
Electron density in (**a**) FS-IGBT, (**b**) HK-IGBT, and (**c**) SIPOS-IGBT and (**d**) carrier density along CC’ in three structures.

Figure 4a–c show the electron density in the drift region of the FS-IGBT, HK-IGBT, and SIPOS-IGBT during the on state. As shown in the figure, VGE was positively biased in the on-state, the electrons accumulated in the SIPOS-IGBT, and a high-density electron accumulation layer was formed along the SIPOS layer. Furthermore, the electron accumulation layer could provide a larger “n-emitter” area for the PiN diode structure in the SIPOS-IGBT, and the ratio of the electron current to the total current, j_e_/j, was increased, resulting in a high injection level at the n-base junction, so the electron density in the drift region was much higher than with the other two structures [31].

**Figure 5:**
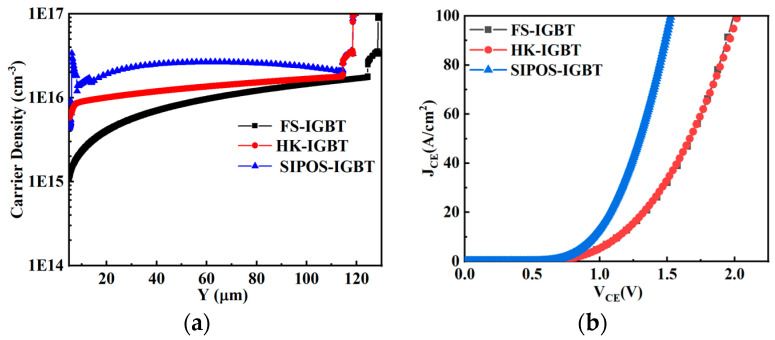
(**a**) Carrier density distribution along A-A’; (**b**) voltage drop at J_CE_ = 100 A/cm^2^.

Due to the charge balance effect, the high-density electrons significantly enhanced the carrier density in the drift region, as shown in Figure 4d and Figure 5a, and with the same collector structure, the carrier density in the SIPOS-IGBT was two times higher than that in the FS-IGBT and HK-IGBT.

With the effects mentioned above, the Von value of the SIPOS-IGBT was significantly reduced compared with the FS-IGBT and the HK-IGBT, as shown in Figure 5b.

**Figure 6:**
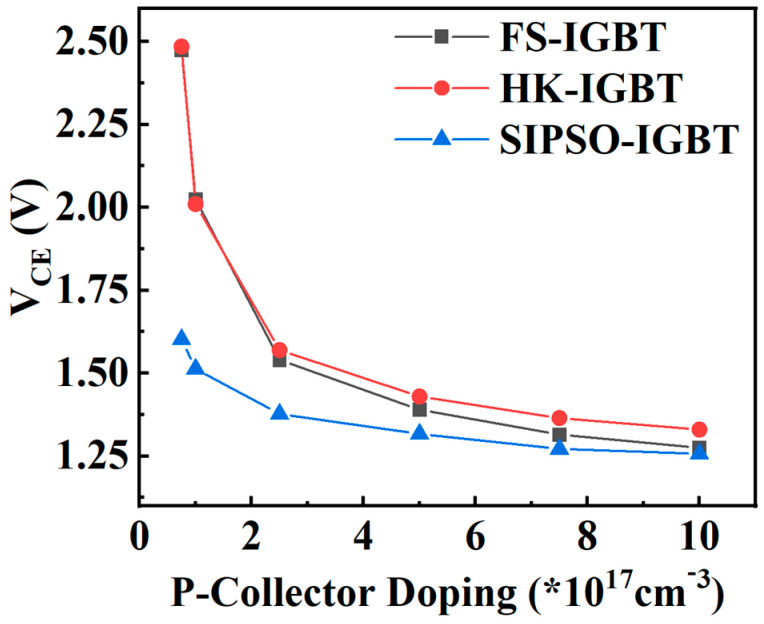
Variation in Von of the FS-IGBT, SIPOS-IGBT, and HK-IGBT with different P-collector doping concentrations.

Figure 6 shows the variation in Von with different P-collector doping concentrations. With the positive-biased V_GE_, the accumulation layers could provide carriers during the on state. Those carriers played an important role when the P-collector doping concentration was low. With a lower P-collector doping concentration, the collector injection efficiency was lower. In the devices whose carriers in the drift region mainly come from the collector injection, such as the FS-IGBT and HK-IGBT, the carrier density was low, leading to a high on-state voltage in the FS-IGBT and HK-IGBT. However, in the SIPOS-IGBT the accumulation layers are the main source of the carriers in the drift region under a low collector injection efficiency, and the accumulated carrier density was much higher than the injected carriers, so the on-state voltage of the SIPOS-IGBT was much lower than that of the FS-IGBT and HK-IGBT.

As the P-collector doping concentration increased, the carriers injected from the collector increased, and the on-state voltage of the FS-IGBT and HK-IGBT decreased with the increase in the P-collector doping concentration. For the SIPOS-IGBT, the carriers introduced by the accumulation layers remained almost constant; with a higher collector injection efficiency, the ratio of the carriers in the drift region related to the accumulation layers decreased; with a high P-collector doping concentration (>8 × 10^17^ cm^−3^), the main source of the carriers in the SIPOS-IGBT became the injected carriers, so the on-state voltage of the SIPOS-IGBT with a high P-collector doping concentration was almost the same as that of the FS-IGBT and HK-IGBT.

### 3.3. Turn-Off Characteristics

SJ-RC-IGBTs employ superjunction structures characterized by charge compensation effects. In the case of a charge balance, the P and N pillars can achieve high doping concentrations under certain blocking capabilities. When the doping concentration of the P and N pillars is high, the conductivity modulation effect caused by hole injection will be affected, which means the static and switching characteristics will also change.

**Figure 7:**
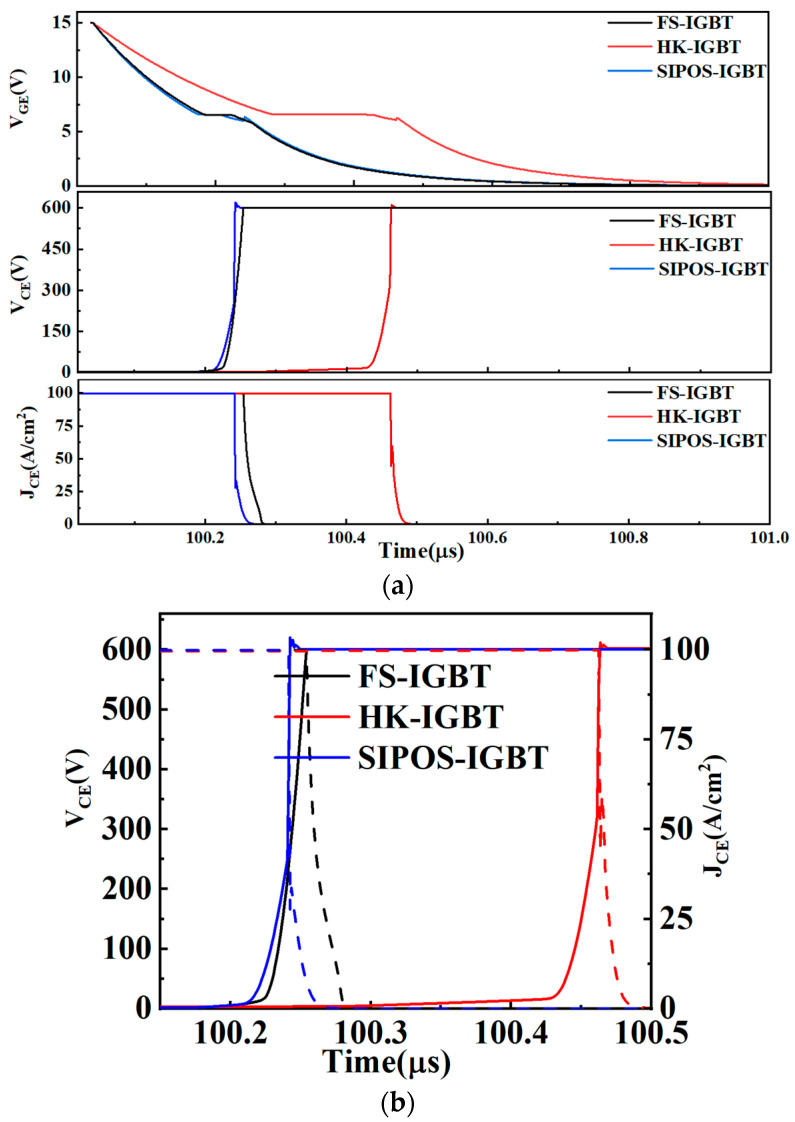
(**a**) Turn-off signals and (**b**) IV curves of the FS-IGBT, SIPOS-IGBT, and HK-IGBT.

Figure 7 shows the turn-off characteristics of the FS-IGBT, SIPOS-IGBT, and HK-IGBT. As shown in the figure, the rising voltage rate of the SIPOS-IGBT and HK-IGBT was different from that of the FS-IGBT, and the rising voltage progress consisted of two stages, namely, a slow stage and a fast stage. In the HK-IGBT, the rising voltage rate of the first stage was lower than that of FS-IGBT and SIPOS-IGBT due to the larger gate capacitance of the HK-IGBT. As shown in Figure 7a, it required more time to charge the capacitance at the first stage of the turn-off progress. This means the HK-IGBT had the longest turn-off delay time (405.8 ns), which was almost twice the turn-off delay time of the FS-IGBT (201.6 ns) and SIPOS-IGBT (210.2 ns); this also limits the high-frequency application of the device.

In the second stage, since the SIPOS-IGBT and the HK-IGBT could deplete the carriers in the drift region from both the vertical direction and the horizontal direction like a super junction structure, the depletion region could be established more quickly with than the FS-IGBT; as a result, a quit fast voltage rising rate was achieved during the fast stage. Furthermore, the concentration of the accumulated carriers in the SIPOS-IGBT decreased immediately as the V_GE_ decreased during the turn-off progress. Compared with the injected carriers, which need to be depleted with an increased V_CE_, the depletion region was established more quickly in the SIPOS-IGBT, in which the carriers in the drift region were dominated by accumulated carriers rather than injected carriers.

**Figure 8:**
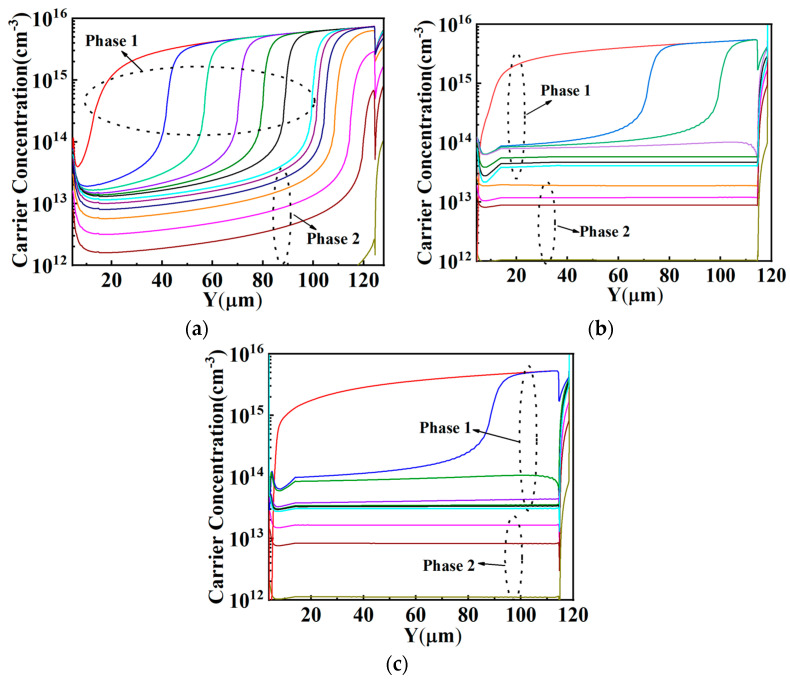
Carrier distribution of the (**a**) FS-IGBT, (**b**) HK-IGBT, and (**c**) SIPOS-IGBT during turn-off transient.

Due to the depletion assistance effect, there was a sharp decrease in the current at the beginning of the decreasing-current stage. As can be seen in Figure 7, the FS-IGBT had a much lower rate of current decrease in the whole turn-off progress, which led to a higher turn-off loss. This phenomenon is explained in Figure 8.

Figure 8 shows the carrier concentration variation during the key part of the turn-off transient (Phase 1: V_CE_ rose from 0 V to 600 V, Phase 2: I_CE_ fell from the load current 100 A/cm^2^ to 0). As shown in Figure 8a, in Phase 1, the space-charge region expanded in the drift region of the FS-IGBT with a constant carrier concentration of about 2 × 10^13^ cm^−3^, which was determined by the current density. After the V_CE_ rose to the bus voltage (600 V), I_CE_ started to fall in Phase 2; at first, the extracted carriers in the drift region supported the current, and the space-charge region kept expanding until the space-charge region reached the FS layer. After the drift region was fully depleted, the current was supported by the remaining carriers in the space-charge region, and the carrier concentration in the space-charge region decreased with time until I_CE_ fell to 0 [32].

Figure 8b,c show the carrier concentration in the HK-IGBT and SIPOS-IGBT. As can be seen in the figure, compared with the FS-IGBT, in Phase 1, the space-charge region expanded to the FS layer before V_CE_ reached the bus voltage due to the depletion assistance effect in the HK-IGBT and SIPOS-IGBT, and the increasing V_CE_ progress turned into a fast stage after the space-charge region reached the FS layer, as shown in Figure 7.

The space-charge region in the HK-IGBT expanded to the FS layer at V_CE_~300 V. In the SIPOS-IGBT, the depletion region expanded to the FS layer at V_CE_~200 V due to quicker space-charge region establishment, as mentioned above. After the space-charge region reached the FS layer, the remaining carriers in the space-charge region supported the load current before the V_CE_ reached the bus voltage. So, in Phase 2, when I_CE_ started to decrease, the remaining carrier concentration in the space-charge region of the HK-IGBT and SIPOS-IGBT was too low to support the high current density, and I_CE_ fell to a small value immediately. Since the space-charge region reached the FS layer at a lower CE voltage in the SIPOS-IGBT, the remaining carrier concentration in the space-charge region of the SIPOS-IGBT was lower than that of the HK-IGBT at the beginning of Phase 2, as shown in Figure 8b,c. The I_CE_ of the SIPOS-IGBT fell to a lower value (about 30 A/cm^2^) faster than that of the HK-IGBT (about 50 A/cm^2^), as shown in Figure 7. After that, the remaining carriers kept decreasing to the doping concentration after I_CE_ fell to 0.

**Figure 9:**
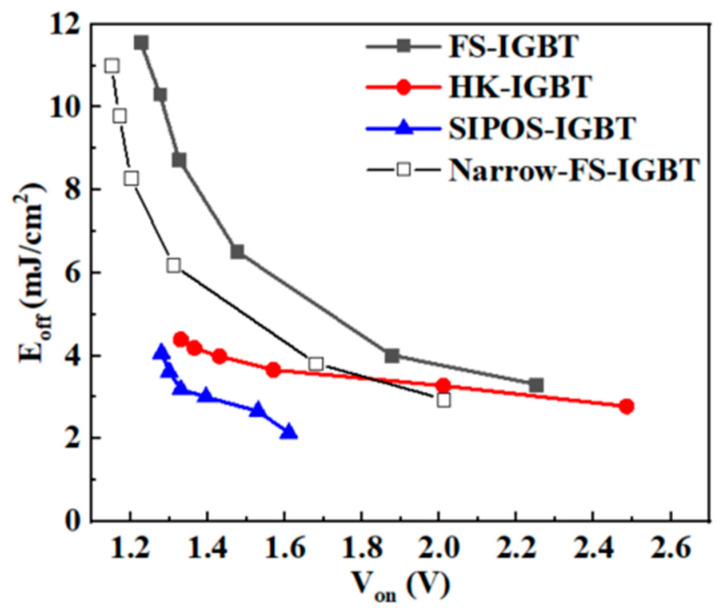
V_on_ vs. E_off_ tradeoff curves for FS-IGBT, HK-IGBT, SIPOS-IGBT, and narrow-mesa FS-IGBT.

As discussed in the prior part, due to the carrier accumulation effect in the SIPOS-IGBT, the required injection efficiency to achieve the same V_on_ was much lower in the SIPOS-IGBT than in the HK-IGBT and FS-IGBT. Since the space-charge region was established quicker in the SIPOS-IGBT, a faster turn-off speed and a lower E_off_ were achieved in the SIPOS-IGBT. As shown in Figure 9, the SIPOS-IGBT obtained a better V_on_ vs. E_off_ tradeoff performance. With the same V_on_ of 1.5 V, the E_off_ of the FS-IGBT, HK-IGBT, and SIPOS-IGBT were 6.4, 3.83, and 2.7 mJ/cm^−3^, and the turn-off loss of the SIPOS-IGBT was 58% lower than that of the FS-IGBT and 30% lower than that of the HK-IGBT. As a comparison, a current commercial FS-IGBT (cell pitch = 1.6 um, with mesa = 0.6 um) was also simulated. Compared with the FS-IGBT with a wider cell pitch, with a narrower cell pitch, the V_on_ was reduced by about 10%, and the turn-off loss was reduced by about 5%. With a V_on_ value of 1.5 V, the E_off_ value of the narrow FS-IGBT was 4.95 mJ/cm^−3^, 46% higher than that of the SIPOS-IGBT. The tradeoff performance was superior to the HK-IGBT in the range (V_on_ > 1.8 V), but the SIPOS-IGBT still had a better performance than the narrow FS-IGBT.

### 3.4. Process Flow

**Figure 10:**
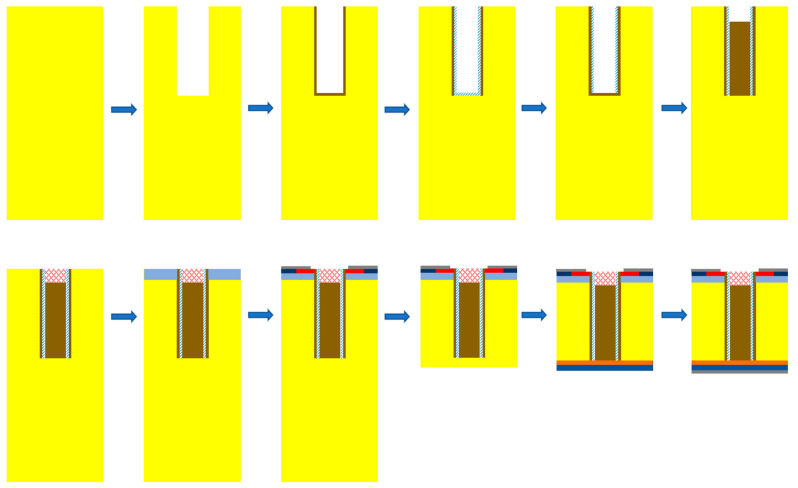
Process flow of SIPOS-IGBT.

As shown in Figure 10, the device fabrication started with a lightly doped N-type (100) Si wafer. A deep trench was etched and followed with thermal oxidation to form the thin oxide layer. Then, the bottom part of the oxide layer was etched, and a thin SIPOS layer was deposited by low-pressure chemical vapor deposition in the trench. The SIPOS layer at the bottom was oxidized by a high-energy laser, and then the achieved oxide layer at the bottom was removed by high-selectivity wet etching. Then, the oxide layer to fulfill the trench was deposited. After that, the oxide was etched back to deposit the poly gate. Then, the N+ emitter and P+ were injected, and thermal annealing was carried out after the formation of the P-body. After the front metal deposition, the wafer thinning process was carried out to achieve a wafer of ~110 um, and the field-stop layer and collector layer were then formed by phosphor or proton injection followed by laser annealing. Finally, the device fabrication was finished after the deposition of the collector metal.

## 4. Conclusions

A novel SIPOS-IGBT is presented in this paper, and the blocking, forward, and switching characteristics were investigated by TCAD simulation. With the SIPOS pillars under the gate electrode, the electric field distribution is modulated to be uniform, which leads to a thinner drift region. Furthermore, electron accumulation layers are formed along the SIPOS layer in the drift region, and these accumulation layers can increase the carrier density and the injection efficiency of the emitter region during the on state. Finally, the carriers in the drift region are thoroughly removed quickly during the turn-off transient. With all these benefits, the V_on_ and E_off_ tradeoff relationship is significantly improved compared with an FS-IGBT and HK-IGBT.

## Figures and Tables

**Table 1:** Key parameters of FS-IGBT, HK-IGBT, and SIPOS-IGBT.

Parameter	FS-IGBT	HK-IGBT	SIPOS-IGBT
Cell pitch/μm	4	4	4
Thickness/μm	120	110	110
N-drift Dop/cm^−3^	5 × 10^13^	5 × 10^13^	5 × 10^13^
Width of N-drift/μm	4	2	2
Width of SIPOS/μm	/	/	0.5
Width of HK/μm	/	2	/
FS layer thickness/μm	5	5	5
FS layer Dop/cm^−3^	1 × 10^16^	1 × 10^16^	1 × 10^16^
Collector thickness/μm	1	1	1
Collector Dop/cm^−3^	1 × 10^17^	1 × 10^17^	1 × 10^17^
K	/	150	/
SIPOS thickness/μm	/	/	0.4
SIPOS resistivity/Ω·cm	/	/	10^10^

## Data Availability

The data presented in this study are available on request from the corresponding author due to (Research project restriction).
